# The necessity of others: Entrepreneurial self-efficacy, TMT collective efficacy, CEO-TMT interface, and entrepreneurial orientation

**DOI:** 10.3389/fpsyg.2023.1095978

**Published:** 2023-03-10

**Authors:** Xiaobao Peng, Xiaofan Song, Emmanuel Mensah Horsey

**Affiliations:** School of Public Affairs, University of Science and Technology of China, Hefei, Anhui, China

**Keywords:** CEO-TMT interface, entrepreneurial self-efficacy, TMT collective efficacy, entrepreneurial orientation, social cognitive theory, entrepreneurial firms

## Abstract

Entrepreneurial orientation is the key factor for enterprises to obtain competitive advantages in dynamic circumstances. Thus, prior studies established the effect of psychological factors, for instance, entrepreneurial self-efficacy on entrepreneurial orientation using social cognitive theory. However, prior studies presented two main opposite views consisting of a positive and negative relationship between entrepreneurial self-efficacy and entrepreneurial orientation as well as providing no alleyway to enrich this relationship. We join the conversation on the positive linkage and argue on the essence of exploring the black box mechanisms to strengthen enterprises’ entrepreneurial orientation. We employed the social cognitive theory and collected 220 valid responses from CEOs and TMTs from 10 enterprises in the high-tech industrial development zones of nine provinces in China to clarify the effect of top management team (TMT) collective efficacy, and CEO–TMT interface on the link between entrepreneurial self-efficacy and entrepreneurial orientation. Our findings show that entrepreneurial self-efficacy positively affects entrepreneurial orientation. In addition, we found that a higher level of TMT collective efficacy strengthens the positive relationship between entrepreneurial self-efficacy and entrepreneurial orientation. Moreover, we discovered differential moderating effects. First, CEO-TMT interface positively affects entrepreneurial orientation when it interacts with TMT collective efficacy and entrepreneurial self-efficacy. Second, CEO-TMT interface has a significant negative indirect effect on entrepreneurial orientation, when it only interacts with TMT collective efficacy. Our study enriches the entrepreneurial orientation literature by positioning TMT collective efficacy and CEO-TMT interface as social cognitive mechanisms underlying the development of entrepreneurial self-efficacy and entrepreneurial orientation nexus. Thus, we open a window of opportunities for CEOs and decision-makers to maintain a sustainable position in the market, grasping more opportunities in uncertain conditions *via* timely entries into new markets and maintaining pre-existing ones.

## Introduction

As a strategic posture at the organizational level (Wales et al., 2021; Ma et al., 2022), entrepreneurial orientation is a central research theme in the field of entrepreneurship (Nakku et al., 2020; Pei et al., 2021; Chew et al., 2022) based on its embodiment of a kind of strategic orientation and decision-making practice in the process of company entrepreneurship (Lumpkin and Dess, 1996; Wales et al., 2021). Entrepreneurial orientation denotes an enterprise’s state or quality behavior that encapsulates the enterprise’s style of making decisions and the practices it adopts to differentiate itself from competitors (Montiel-Campos, 2018). In the entrepreneurship literature, the concept of entrepreneurial orientation has been addressed from two main standpoints (Rauch et al., 2009; Covin and Wales, 2012; Wales et al., 2013; Martens et al., 2016). The first emphasizes entrepreneurial orientation as a unidimensional concept composed of innovativeness, risk-taking, and proactiveness, which must positively vary together for an entrepreneurial orientation to be manifested (Miller, 1983; Covin and Slevin, 1989). In line with Miller (1983) and Covin and Slevin (1989)’s conceptualization, Anderson et al. (2015) proposed two dimensions encompassing the joint exhibition of entrepreneurial behaviors (innovativeness and proactiveness) and a managerial attitude or inclination (risk-taking) that jointly represent the higher-order entrepreneurial orientation construct. The second emphasizes entrepreneurial orientation as a multidimensional concept with two additional dimensions: competitive aggressiveness and autonomy (Lumpkin and Dess, 1996). The multidimensional approach proposes that the dimensions of entrepreneurial orientation may manifest in different combinations, each representing a distinct and independent aspect of entrepreneurial orientation. Albeit the two conceptualizations of entrepreneurial orientation recent meta-analyses indicated that the unidimensional approach by Miller (1983) and Covin and Slevin (1989) is the predominant and in general have explained entrepreneurial firms better performance than firms that are not entrepreneurial orientation firms or conservatively managed (Montiel-Campos, 2018).

The broad influence of entrepreneurial orientation is revealed in its tendency to encourage enterprises to explore potential market opportunities (Tasavori et al., 2018; Sellappan and Shanmugam, 2020) and thus contributes to enterprise performance (Lumpkin and Dess, 2001; Wales et al., 2013; Diaz and Sensini, 2020). In light of its crucial role in enterprises’ entrepreneurial processes, prior studies explored the impact of personal cognitive factors such as entrepreneurial self-efficacy (Chaston and Sadler-Smith, 2012; Hwan, 2020). Entrepreneurship researchers defined entrepreneurial self-efficacy as the measure of an individual’s belief in his or her capability to successfully set up an entrepreneurial venture (McGee et al., 2009) and thus emphasizes tasks execution such as innovation, management, finance, and marketing that are critical in new venture formation (Hsu et al., 2017). In particular, entrepreneurial self-efficacy is one of the important dimensions of entrepreneurial cognition, which plays an indispensable role in enterprises’ new business opportunities discovery, entrepreneurial strategic decisions making, and behaviors (Jiatong et al., 2021; Neneh, 2022), has been repeatedly endorsed as a key determinant of entrepreneurial orientation.

However, prior studies explored different hypothetical perspectives (e.g., individual differences, and entrepreneurial environment), theories (e.g., social learning theory, social cognitive theory) and presented differential empirical validations (e.g., positive and negative) of entrepreneurial self-efficacy on entrepreneurial orientation, leading to no single consensus of its impact. For example, existing studies found both positive (Poon et al., 2006; Eniola, 2020; Seet et al., 2020), and negative relationships (Chandler and Jansen, 1997; Markman et al., 2002) as well as no direct relationship (Kolvereid and Isaksen, 2006; Boukamcha, 2015) between entrepreneurial self-efficacy and entrepreneurial orientation (see Table 1). Cumulatively, the inconsistent findings suggest that the impact of entrepreneurial self-efficacy on entrepreneurial orientation is highly context-dependent. In this light, social cognitive theory (SCT) serves as the foundation to understand how its hypothesis on human agency, perceptions, motivations, and choices of individuals and teams (Alavi and McCormick, 2018; Donohoo et al., 2018) could explain other factors in the nexus between entrepreneurial self-efficacy on entrepreneurial orientation. In other words, additional examinations are needed to uncover the black-box mechanisms pivotal to strengthening the positive effect or resolving the negative effect of entrepreneurial self-efficacy on entrepreneurial orientation.

**Table 1:** Summary of studies linking entrepreneurial self-efficacy and entrepreneurial orientation.

Theoretical approach	Author (date)	Sample, variables, and measures	Results
Social Cognitive Theory	Poon et al. (2006)	Engaged 96 SMEs entrepreneurs	Generalized self-efficacy has a positive role in promoting entrepreneurial orientation.
		Measured generalized self-efficacy using 10 items scale (Sherer et al., 1982) and entrepreneurial orientation with nine items scale (Miller, 1983; Covin and Slevin, 1989).	
	Hwan (2020)	Engaged 440 students from China and South Korea	Entrepreneurial self-efficacy has positive relationship with entrepreneurial orientation.
		Measured entrepreneurial self-efficacy using 5 items scale (Chen et al., 1998) and entrepreneurial orientation with 9 items scale (Covin and Slevin, 1989).	
	Mohd et al. (2015)	Engaged 162 SMEs in manufacturing in all Malaysian states	Self-efficacy positively correlates with entrepreneurial orientation.
		Measured self-efficacy using 22 items scale (Chen et al., 2004) and entrepreneurial orientation with 12 items scale (Lumpkin and Dess, 1996).	
	Chandler and Jansen (1997)	Retrieved samples from the State of Utah records of business incorporation	Self-efficacy does not predict subsequent entrepreneurial performance.
		Measured self-efficacy (entrepreneurial efficacy, managerial efficacy, and technical efficacy) using Chandler and Jansen (1992) scale and entrepreneurial performance using sales/earnings and growth.	
	Seet et al. (2020)	Engaged 204 South Australian SME founders	Entrepreneurial self-efficacy positively correlates with the three dimensions of entrepreneurial orientation (innovation, risk-taking, proactiveness).
		Measured entrepreneurial self-efficacy using 4 items scale (Zhao et al., 2005) and entrepreneurial orientation with 9 items scale (Covin and Slevin, 1989).	
Achievement Goal Theory	Boukamcha (2015)	Engaged 240 participants in four business incubators	In the absence of motivation and enthusiasm, self-efficacy has no significant effect on entrepreneurial intention.
		Measured perceived self-efficacy using 6 items scale (Chen et al., 2001) and entrepreneurial intention with 6 items scale (Gundry and Welsch, 2001).	
Social Learning Theory	Li and Liao (2014)	Engaged 70 traditional enterprises and 92 Hi-tech enterprises	Entrepreneurial self-efficacy has a positive impact on entrepreneurial orientation.
		Measured entrepreneurial self-efficacy using McGee et al. (2009) scale, Sherer et al. (1982) efficacy scale, and entrepreneurial orientation with 9 items scale (Covin and Slevin, 1989).	
Planned Behavioral Theory and Reasoned Action Theory	Kolvereid and Isaksen (2006)	Engaged 297 Norwegian business founders	Self-efficacy had no significant influence on entrepreneurial behavior.
		Measured entrepreneurial self-efficacy using 18 items scale (Betz and Hackett, 2006) and self-employment with 4 items scale (Gundry and Welsch, 2001).	
Disadvantage Theory	Markman et al. (2002)	Engaged 217 patent inventors	Self-efficacy can negatively affects entrepreneurial experience and indirectly affect entrepreneurial decision-making.
		Measured general self-efficacy using 8 items scale (Eden and Aviram, 1993; Chen et al., 2001) and regretful thinking in quantitative, qualitative and magnitude, including an open-ended question and a seven-point scale.	

With this objective, the present study attempts to explore “what conditions” reinforce the effect of entrepreneurial self-efficacy on entrepreneurial orientation. We argue that the internal decision-making and entrepreneurial-oriented behavior of any entrepreneurial enterprise are inseparable from the unique role of the CEO’s entrepreneurial self-efficacy and other TMT members (Miao et al., 2019). Specifically, we argue that although a CEO’s characteristics such as high entrepreneurial self-efficacy can influence entrepreneurial success, the CEO’s performance also depends on the characteristics of the group he works with. Consistent with the works of Hwan (2020) and (Mohd et al., 2015), we employ the theoretical hypothesis of the social cognitive theory (SCT) but point out TMT collective efficacy as a collective concept (alleyway or mechanism) that complements the self-efficacy of the CEO, thereby further impacting entrepreneurial orientation. TMT collective efficacy has its root in collective efficacy that denotes a team or group’s members’ beliefs in their agentive abilities to tackle challenges and execute tasks successfully (Bandura, 1997) which is contingent upon team motivation (Lewis, 2011) and awareness of collective cognition action (Gibson and Earley, 2007). Recent studies put forward the concept of TMT collective efficacy to capture a top management team’s conviction in their joint or collaborative capabilities to organize and execute the course of action (team processes) to actualize expected levels of attainments (outcomes; Luo and Lin, 2022). Furthermore, it plays a cornerstone role in shaping the enterprise strategy process and organizational results (Luo and Lin, 2022). In the face of a complex market environment, the TMT with high collective efficacy has stronger rational thinking ability and tends to set clearer goals, thereby helping the CEO to make more effective entrepreneurial-oriented strategic decisions (Elms et al., 2022; Goswami, 2022).

In this regard, the concept of CEO-TMT interface sums up the connection between the CEO and TMT and its role in entrepreneurial decision-making and performance. CEO-TMT interface refers to the profound impact a CEO can have on shaping the attitudes and behaviors of the TMT that consequently affects the enterprise-level outcomes (Ou et al., 2018). Researchers asserted that TMT is a potential source of critical strategic resources that acts as an essential driving factor in entrepreneurial-oriented strategic decisions. Other studies showed that CEO is the leadership core of the TMT and a decisive factor that determines strategic choices, performance (Ou et al., 2018), and enterprise development (Chen et al., 2022). In this vein, CEOs and TMTs form collective interactions and dependencies (Hambrick et al., 2015). Prior studies have shown that the connection between the CEO and the other members of TMT helps tackle uncertainties and conflicts (differences in strategic decisions) *via* collective tasks such as risk perceptions and innovation decisions. Therefore, the interaction between the two has a significant impact on business practices and all aspects of business operations (Qiao et al., 2021). In an enterprise with a strong CEO-TMT interface, TMT members can exert their own values and talents, possess high-efficiency beliefs, and can gather collective strength to accomplish corporate goals. In addition, the interaction between the CEO and TMT can improve the quality of information obtained by both parties, reduce information asymmetry, promote high-quality discussion and understanding of strategy formulation, and achieve better internal decision-making consistency (Georgakakis et al., 2022), thereby influencing entrepreneurial-oriented strategic decision-making. Based on this, we believe that the influence of a CEO’s entrepreneurial self-efficacy on entrepreneurial orientation can be influenced by the moderating roles of TMT collective efficacy and CEO-TMT interface, hence, strengthening the relationship.

Our study has two core theoretical implications. Firstly, this study focuses on the combined effects of CEOs and TMT members, to provide new insight, to resolve the inconsistency of existing research conclusions about the relationship between entrepreneurial self-efficacy and entrepreneurial orientation. Some studies showed that entrepreneurial self-efficacy has a significant impact on entrepreneurial orientation. However, most studies only focused on CEOs’ entrepreneurial self-efficacy in the sampled enterprises. This study explores the effect of TMT collective efficacy on the relationship between entrepreneurial self-efficacy and entrepreneurial orientation, the results show that entrepreneurial orientation is affected not only by the CEO’s entrepreneurial self-efficacy but also by the collective impact of the entire TMT. This finding allows scholars to fully understand the drivers of entrepreneurial orientation. Secondly, this study expands and supplements research on the relationship between entrepreneurial self-efficacy and entrepreneurial orientation from the perspective of CEO-TMT interface. Considering the individual differences and the operational mechanism of behavioral integration within TMT, we point out that the CEO–TMT interface also has an important impact on entrepreneurial behavior, which has more explanatory power than previous studies.

## Review of relevant research

A considerable number of studies confirmed the positive influence of CEOs’ personal cognitive factors on enterprise strategic decision-making (Fernández-Pérez et al., 2016). Moreover, scholars increasingly paid attention to the cultivation of the CEO’s cognitive ability and the role of the CEO’s entrepreneurial cognition in the process of entrepreneurship. In the field of entrepreneurship, entrepreneurial self-efficacy is a dimension of entrepreneurial cognition, which reflects the CEO’s confidence or belief that his/her entrepreneurial behavior affects his/her environment or achieves the goals and results of entrepreneurial behavior (Chen et al., 1998; Hand et al., 2020). Most of the theoretical and empirical studies on corporate entrepreneurship in the existing literature believed that entrepreneurial self-efficacy can positively influence entrepreneurial orientation and promote enterprises to continuously enhance their competitive advantages in key business areas (Poon et al., 2006; Eniola, 2020; Seet et al., 2020). Thus, a positive correlation exists between entrepreneurial self-efficacy and entrepreneurial orientation. To this effect, entrepreneurial self-efficacy is an important cognitive trait that predicts entrepreneurial orientation.

An array of empirical studies, for example, Poon et al. (2006) demonstrated a positive correlation between generalized self-efficacy and entrepreneurial orientation in an empirical study of 96 SME entrepreneurs. In recent years of research, Seet et al. (2020) conducted a stratified random sampling survey of 204 early-stage South Australian micro and small business founders. The results showed that a significant positive correlation exists between entrepreneurial self-efficacy and entrepreneurial orientation. Moreover, a stronger entrepreneurial self-efficacy would lead to a higher entrepreneurial orientation. Specifically, entrepreneurial self-efficacy is positively correlated with innovation, proactiveness, and risk-taking. Comparatively, individuals with low self-efficacy tend to avoid building competencies or taking risks, whereas those with high self-efficacy instill perseverance, effort, and confidence in individuals, making them more confident that their own business is feasible (McGee and Peterson, 2019; Newman et al., 2019), to make entrepreneurial decisions in line with entrepreneurial-oriented activities. In other words, entrepreneurial self-efficacy can increase the efforts, perseverance, and confidence of CEOs, and improve the efficiency of their decision-making. Entrepreneurial self-efficacy can also effectively cope with the change of thinking mode and emotional response brought by changes in the external environment, thereby improving entrepreneurial orientation (Bandura and Walters, 1977). Strongly driven by high entrepreneurial orientation, entrepreneurial self-efficacy can encourage CEOs to continue to work efficiently in complex decision-making scenarios. In addition, it can strengthen an individual’s sense of control over the results of entrepreneurial behavior, to take positive decisions and receive positive feedback. This case is conducive for CEOs to innovate and improve existing products or services ahead of competitors, identify and develop new business opportunities, and actively take on more and larger entrepreneurial risks. Moreover, they can actively undertake more and larger entrepreneurial risks, thereby making the implementation of entrepreneurial-oriented strategies more effective.

Table 1 summarizes the results of relevant studies. The existing literature mainly supported the empirical evidence that a positive correlation exists between entrepreneurial self-efficacy and entrepreneurial orientation. However, other studies believed that the relationship between entrepreneurial self-efficacy and entrepreneurial orientation is irrelevant, nonlinear, or even negatively correlated. Kolvereid and Isaksen (2006) empirically studied 297 Norwegian business founders and found that there is no correlation between self-efficacy, entrepreneurial behavior, and entrepreneurial intention. The main reason is that compared with other countries, Norway had a simple, tolerant and friendly entrepreneurial environment. In addition, self-efficacy measures are less specific. Several studies showed that entrepreneurs with a high sense of entrepreneurial self-efficacy and a high degree of optimism have a stronger ability to execute entrepreneurial behaviors, but high self-efficacy can lead to “overconfidence” and deviation in strategic decisions (Hmieleski and Baron, 2008). Additionally, Markman et al. (2002) confirmed that entrepreneurial self-efficacy can increase the negative emotions brought by entrepreneurial experience, increase the psychological pressure on entrepreneurs, and indirectly affect entrepreneurial decision-making, thus adversely affecting entrepreneurial orientation.

In the literature, inconsistent results on the relationship between entrepreneurial self-efficacy and entrepreneurial orientation exist. Besides, the literature on the mechanisms of TMT collective efficacy and CEO–TMT interface is limited, particularly in the context of Chinese entrepreneurial culture and the influence of entrepreneurial self-efficacy on entrepreneurial orientation. In sum, insight into the mechanisms is still a “black box” that has not been uncovered.

## Hypothesis development

### The moderating effect of TMT collective efficacy

A team is a dynamic organizational structure, and everyone in the team depends to some extent on cooperation with others to complete work tasks (Alavi and McCormick, 2018). The increasing interdependence of human functions encourages the role of collective subjectivity (Bandura, 2000). Collective efficacy is a subjective understanding of team members’ abilities, and it is the common belief of team members that the team can successfully complete a specific task (Chen et al., 2019; Luo and Lin, 2022). Once collective efficacy enters the belief structure of TMT members, it has a significant impact on individual and team entrepreneurial behavior (Srivastava et al., 2006). In this process, collective efficacy reflects the organizational commitment and responsibility of TMT members to the team and influences individual behaviors in the team (Chen et al., 2019). These include individual’s perceptions and attitudes toward entrepreneurship, how much effort they put into performing group tasks, how well they can work with team members to make the right decisions, and how well they can maintain their previous level of effort when collective efforts fail to achieve goals quickly or when they face opposition (Gibson and Earley, 2007; Donohoo et al., 2018). This view also predicts that high levels of entrepreneurial-oriented behavior are based on TMT collective efficacy.

Social cognitive theory emphasizes the role of psychological cognitive states (Bandura, 2001). From the psychological level, collective efficacy is the effective coordination and integration of self-efficacy (Bandura, 1997). Since the CEO has absolute control and dominance over the team resources, most of the strategic decisions related to the survival and development of the company are made by the CEO (Zhong et al., 2022), and the CEO’s decisions are implemented through the TMT members (Li and Jones, 2019). In this case, on the one hand, entrepreneurial-oriented behaviors are decisions and actions made under the CEO’s bounded rationality. Due to the limitations of his prior experience and cognitive level, the decision-making process also requires the participation of TMT members. TMT collective efficacy can minimize the apathy, passivity, and inaction of TMT (Bandura et al., 1999; Luo and Lin, 2022), while improving their decision-making ability (Donohoo et al., 2018). In a TMT with high collective efficacy, the CEO believes in himself and the enterprise’s ability to achieve the desired results (Chen et al., 2019). Thus, they will constantly follow the market trail, remain vigilant of competitors, seize new market opportunities ahead of competitors, and launch innovative products and services (Elms et al., 2022). On the other hand, in an enterprise, if TMT members have a strong sense of collective efficacy as well as collective power and faith (Alavi and McCormick, 2018), then TMT members can substantially impact their entrepreneurial activity. In particular, faith will stimulate the confidence of the CEO to successfully play the entrepreneurial role and complete the entrepreneurial task. Also, to make decisions in favor of entrepreneurial orientation activities.

However, entrepreneurship is not always successful (Tasa and Whyte, 2005; Wynn and Jones, 2019). TMT characterized by high collective efficacy are more likely to recover from failure and are more likely to create a positive work environment and emotional climate (DeRue et al., 2010; Rapp et al., 2021). This is because the positive beliefs brought by TMT collective efficacy can inhibit and alleviate this failure cognition, thus effectively controlling the team’s anxiety and reducing the negative emotions associated with the execution of corporate tasks (Elms et al., 2022). In this case, the executive team tends to view the situation as an opportunity full of positivity and development, rather than complete negativity and danger. CEOs are also more willing to take risks associated with active competitive behavior and are more willing to redo Innovative thinking (Knight et al., 2001), always stick to the task direction, and implement entrepreneurial-oriented decision-making. Based on this, we propose that:

*H1*: TMT collective efficacy positively strengthens the relationship between entrepreneurial self-efficacy and entrepreneurial orientation.

### The moderating role of CEO–TMT interface

As a psychological perception with strong subjectivity, TMT collective efficacy is mainly generated from the interdependence and interaction of TMT members (Banks et al., 2014; Chen et al., 2019). Research has shown that the most effective interactions occur between the most powerful member of the team (the CEO) and other members of TMT. In the management of the TMT, the CEO should not only pay attention to whether the corporate goals can be achieved, but also whether he or she and other members of the TMT can communicate and support each other (Herdman et al., 2017). Therefore, CEO-TMT interface is particularly important (Lin and Lin, 2019). CEO-TMT interface refers to the dynamic process in which the CEO and other members of TMT connect and interact with each other (Georgakakis et al., 2022). It is the opposite of a state of “either I or he.” Such interaction can only be achieved when the team presents a kind of mutual identification and integration (Qiao et al., 2021).

Due to the limited personal ability and resources of the CEO, it is impossible to comprehensively identify and judge the internal conditions and external environment of the enterprise (Hambrick, 2007). Differences in the characteristics of the CEO and other members of TMT will lead to different values and cognitive behaviors of team members, which will affect the perspective of TMT members on the problem and the way to deal with the problem. CEO-TMT interface is the result of their interaction (Simsek et al., 2018). Although the shared beliefs of team members increase when TMT collective efficacy comes into play, the CEO and other members of TMT are also prone to an “us-them” adversarial situation. At this time, serious communication problems and conflicts often occur within the TMT (Bezrukova et al., 2012), which can weaken the TMT’s collective efficacy and reduce the quality of decision-making.

**Figure 1:**
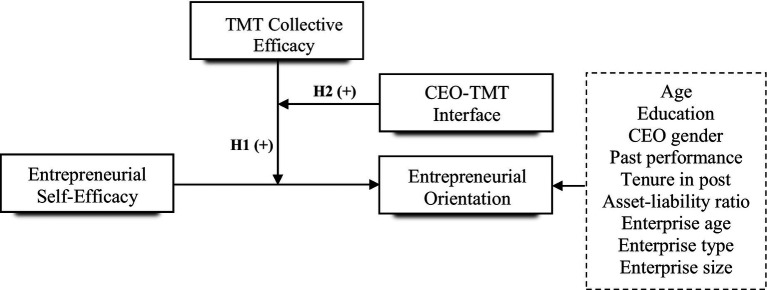
The hypothesized model.

Prior research underscored that a successful TMT requires effective interaction between members, especially between the CEO and members of the TMT (Bachrach et al., 2022). On the one hand, as the interaction between the CEO and other members of TMT becomes stronger and stronger, the information exchange among members will increase and show a diversified pattern (Herdman et al., 2017), and the emotional conflict between members will gradually decrease (Yang et al., 2021). TMT collective efficacy will be significantly improved. On the other hand, the higher the degree of similar interaction between the CEO and other members of TMT, the more they agree with each other (Buyl et al., 2017), and the risk conflict, innovation conflict, and cognitive conflict between the two will be reduced, thereby promoting the integration of team behavior (Li and Liao, 2014) and improving the efficiency of decision-making (Lin and Lin, 2019), which has a positive impact on entrepreneurial orientation (Figure 1). Thus, we propose that:

*H2*: CEO-TMT interface positively moderates the moderating effect of TMT collective efficacy on the relationship between entrepreneurial self-efficacy and entrepreneurial orientation.

## Methods

### Participants and procedure

To evaluate the hypotheses of this study, we adopted a quantitative analysis method. Considering that there are no secondary public data for our selected variables, such as enterprise team and operation mechanism, this study adopted the questionnaire survey technique to collect data which is a valid prior research practice (Li et al., 2021; Chew et al., 2022; Xiabao et al., 2022). Cross-sectional surveys in China have mainly necessitated the translation of the original (English) questionnaire to simplified Chinese or Mandarin to help participants fully grasp questions and provide answers that represent their experiences or phenomenon of their enterprises (Xiabao et al., 2022). Borrowing insights from past approaches (Brislin, 1970), a team of bilingual translators performed a back-translation which consisted of an iterative procedure of repeated independent translation and back-translation and engaged different independent bilingual translators (Triandis and Brislin, 1984). Bilingual translators translated each instrument/item from the original English language to the Chinese language. Errors discovered were repeatedly checked until the team was convinced of a good similarity (concept equivalence) and the absence of grammatical problems between the two versions of the questionnaire. The questionnaire had three parts. The first part comprised control variables to help gather information on the samples’ demographic statistics. The second and third parts comprised widely used and well-validated measurement scale items retrieved from past studies, for instance, the works of Luo and Lin (2022) and Georgakakis et al. (2016). The items assessed our sample on the main constructs (see the measures section). The preliminary sampling phase focused on soliciting respondents’ feedback to confirm the suitability and clarity of the Chinese version of the questionnaire as well as ascertain whether the questions met the expected outcomes per their enterprises’ activities. For instance, participants were provided with definitions and brief descriptions of entrepreneurial orientation. Afterward, final concept equivalence checks were made to the Chinese questionnaire and administered to the participants through email and WeChat, a Chinese social media application. To encourage response, after sending out the questionnaires, we arranged for special personnel to make follow-up calls and serve reminders to facilitate respondents’ cooperation. In addition, we set “trap questions” in the questionnaire, such as “please select the second option from the left,” to ascertain whether the respondents were attentive and had a keen interest in the survey.

We chose China to conduct the research survey because China is a country with active entrepreneurship, fast economic growth, and is recognized as having a rapidly rising entrepreneurial ecosystem of domestic enterprises. In addition, considering the differences in the level of economic development and marketization among different regions in China, the levels of innovation and entrepreneurship are different to a certain extent. Therefore, some Chinese representative regions are eligible samples for research.

We engaged 10 CEOs and 210 TMTs from 10 enterprises, totaling 220 samples. The sample was drawn from a database of 1,000 enterprises in the high-tech industrial development zones of nine provinces in the Yangtze River Delta (Shanghai, Jiangsu, Anhui, Zhejiang), Pearl River Delta (Guangdong, Shenzhen), Bohai Rim (Shandong), Central China (Henan), and northwest China (Shanxi). This research collected the data through two main channels. First, we got in touch with the market supervision bureaus and tax authorities of each province and sent questionnaires to the corresponding entrepreneurial enterprises (e.g., intellectual property demonstration enterprises) through the market supervision bureaus and tax authorities of 16 provinces. Second, through an existing relationship network, we liaised with acquaintances to contact the senior management of the enterprises and sent questionnaires after their consent. The respondents of the questionnaire included CEOs and TMTs of the enterprises. Although we had less participation, all 220 (out of 1,000 copies of the questionnaire distributed) responses gathered were logical. This represents a 22% recovery rate.

### Measures

To ensure the validity and reliability of the measurement tool, this study used the scales of high-quality academic journal publications (Luo and Lin, 2022) and made it conform to the Chinese context according to the suggestions of relevant professionals. In the survey, to avoid the likelihood of common method bias caused by self-reported data, the items of entrepreneurial self-efficacy were answered by TMT members while items of TMT collective efficacy were addressed by the CEO. The items measuring CEO–TMT interface and entrepreneurial orientation were addressed jointly by each enterprise’s CEO and TMT members. All items were scored on a 5-point Likert scale, with 1—strongly disagree and 5—strongly agree.

### Entrepreneurial orientation

We measured entrepreneurial orientation based on Miller (1983),s conceptualization with the modifications introduced by Covin and Slevin (1989) and Covin and Miller (2014). Consistent with these studies, we also employed entrepreneurial orientation as a unidimensional construct involving three dimensions with nine items: innovation (measured with items EO1–EO3); proactivity (items EO4–EO6); and risk-taking (items EO7–EO9). This scale captures an enterprise’s entrepreneurial orientation-related attitude and has been frequently used as well as received high reliability and validity ratings (Covin and Wales, 2012). The Cronbach’s alpha (α) coefficient of the scale was 0.965.

### Entrepreneurial self-efficacy

For the measurement of entrepreneurial self-efficacy, this study utilized the 10 measurement items of Schwarzer et al. (1997) and McGee et al. (2009). We asked the participants to evaluate their perceptions of various behaviors, which are robust predictors of enterprise performance (Miller, 1983). Examples of items include: I was able to provide new ideas for existing products or services. The Cronbach’s alpha (α) coefficient of the scale was 0.949.

### TMT collective efficacy

We measured TMT collective efficacy with four items adapted from Luo and Lin (2022). Sample items include: Our TMT is capable of handling heated competition with direct rivals. The Cronbach’s alpha (α) coefficient of the scale was 0.817.

### CEO–TMT interface

The research project of Georgakakis et al. (2016) mainly discussed the moderating effect of CEO–TMT interface from five dimensions: career experience, political background, educational background, functional background, and social experience. There were five items in total. Sample items include: CEO and TMT share similar common tenure. The Cronbach’s alpha (α) coefficient of the scale was 0.915. Table 2 shows the reliability and validity of these variables.

**Table 2:** Results of factor analysis.

Construct	Indicators	Factor loadings	Cronbach’s alpha (α)	Average variance extracted (AVE)	Composite reliability (CR)
Entrepreneurial orientation	EO1	0.893	0.965	0.623	0.941
	EO2	0.915			
	EO3	0.833			
	EO4	0.915			
	EO5	0.784			
	EO6	0.920			
	EO7	0.819			
	EO8	0.882			
	EO9	0.876			
Entrepreneurial self-efficacy	ESE1	0.851	0.949	0.583	0.840
	ESE2	0.988			
	ESE3	0.669			
	ESE4	0.599			
	ESE5	0.994			
	ESE6	0.573			
	ESE7	0.744			
	ESE8	0.603			
	ESE9	0.988			
	ESE10	0.717			
TMT collective efficacy	TCE1	0.954	0.817	0.678	0.911
	TCE2	0.633			
	TCE3	0.478			
	TCE4	0.891			
CEO*-*TMT interface	CTI1	0.647	0.915	0.760	0.966
	CTI2	0.962			
	CTI3	0.682			
	CTI4	0.969			
	CTI5	0.801			

### Control variables

We controlled for CEO gender, age, education, past performance, tenure in post, and asset-liability ratio as well as enterprise age, size, and type based on their possible influence on the occurrence of entrepreneurial orientation. CEO gender was controlled following the prior notion that it relates to entrepreneurial enthusiasm which can decrease with the age of CEOs (Levesque and Minniti, 2006). Similarly, controlling for the CEO’s educational level is consistent with the belief that it affects their entrepreneurship activities (Hamilton, 2012). Scholars also found that the past performance of an enterprise can increase the scale of redundant resources to shape innovative and entrepreneurial activities (Zahra et al., 2000). Enterprise age may affect the number of resources and opportunities that an enterprise obtains (Salunke et al., 2013) and consequently affects entrepreneurship. In addition, the asset-liability ratio of enterprises largely determines the ability of enterprises to obtain external resources, which can also affect entrepreneurial activities. Based on the resource-based theory, entrepreneurial orientation is driven by innovation, which generates resource demands in the process of innovation and drives enterprises to acquire different resources. Therefore, when the types of enterprises (high-tech and non-high-tech companies) are inconsistent, the impact on entrepreneurial direction is also different (Huang and Wang, 2013). We operationalized the control variables following extant studies (David et al., 1998; Zahra et al., 2000; Sinatoko Djibo et al., 2022; Xiabao et al., 2022). Accordingly, CEO gender was coded with 1 = representing male and 2 = representing female. Age was coded with values 1 to 4 (e.g., 1 = 25 and below; 2 = 26 to 35; 3 = 36 to 45; 4 = 46 and above; Xiabao et al., 2022). Past performance was operationalized as the 3-year average return on the enterprise assets or the net earnings divided by its assets (David et al., 1998; Zahra et al., 2000). Tenure in post was operationalized using the number of years the CEO has been served in his or her current rank or position (Simsek, 2007). Enterprise size was measured using the natural logarithm of the total number of employees of the enterprises (Casillas et al., 2010). Asset-liability ratio was operationalized as the aggregate of the enterprise’s existing financed loans (accrued and unpaid). We operationalized enterprise age using 1 = equal to or less than 4 years and 2 = otherwise and enterprise size with 1 = implying small; 2 = medium; and 3 = large (Sinatoko Djibo et al., 2022). Enterprise type was assessed using 1 = implying high-tech and 2 = non-high-tech companies (Huang and Wang, 2013).

## Data analysis and results

### Reliability and validity analysis

This study’s constructs/items reliability and validity estimation aligns with extant studies conducted in China (Lewis, 2017; Sinatoko Djibo et al., 2022) and abroad (Luo and Lin, 2022). Accordingly, SPSS-AMOS statistical procedures were employed. We conducted a sampling adequacy test using the Kaiser–Meyer–Olkin Measure (KMO) technique. The KMO score of 0.867 was higher than the minimum acceptable value of 0.7 (Kaiser, 1974). This indicated that the sample size of this study was sufficient. Next, we conducted an exploratory factor analysis (EFA) to decipher constructs/items’ reliability and validity. At the items level, all factor loadings were above the threshold of 0.6 for significance (Hair et al., 2017). The constructs/variables level validity analysis was estimated using Cronbach’s alpha (α), composite reliability (CR), and average variance extracted (AVE) tests. For each of the tests (see Table 2), all the Cronbach’s alpha (α) and composite reliability (CR) values were greater than the recommended benchmark of 0.7 (Hair et al., 2017). The AVE scores for all constructs were higher than 0.50 (ranging between 0.583 to 0.760; Fornell and Larcker, 1981). For discriminant validity, we calculated the square root of constructs AVEs reported in Table 2. As shown in Table 3, the square root of AVEs aligned with the given cut-off margin of not lesser than 0.7 for validity. Additionally, we employed structural equation modeling using SPSS-AMOS software to test our model’s goodness of fit to the data. The results: χ^2^/df, NFI, CFI, RMSE, and IFI were 2.136, 0.911, 0.950, 0.072, and 0.951, respectively, showed a good fitting degree with the data (Lewis, 2017; Sinatoko Djibo et al., 2022).

**Table 3:** Means, standard deviation, and bivariate correlations for all variables.

Variable	Mean	S.D	1	2	3	4	5	6	7	8	9	10	11	12	13
1. Age	40.890	5.114	1												
2. Education	2.030	0.457	0.222^**^	1											
3. CEO gender	0.790	0.408	−0.014	−0.116	1										
4. Past performance	10.300	5.670	0.374^**^	−0.061	0.029	1									
5. Tenure in post	6.650	4.414	0.143^*^	0.242^**^	−0.125	0.303^**^	1								
6. Asset-liability ratio	2.420	1.567	−0.067	−0.029	−0.133^*^	0.047	−0.052	1							
7. Enterprise age	8.270	2.477	0.232^**^	−0.006	0.092	0.256^**^	0.281^**^	0.088	1						
8. Enterprise type	0.205	0.404	0.199^**^	−0.030	0.039	0.171^*^	−0.272^**^	0.108	0.137^*^	1					
9. Enterprise size	3.020	1.145	0.030	−0.019	0.108	0.171^*^	−0.073	0.091	0.275^**^	0.227^**^	1				
10. ESE	4.255	0.536	−0.005	0.072	0.182^**^	0.032	0.039	−0.021	−0.032	0.039	0.151^*^	**(0.916)**			
11. TCE	4.286	0.519	0.036	−0.014	0.015	0.109	0.109	0.130	−0.036	0.008	0.051	0.331^**^	**(0.954)**		
12. CTI	4.000	0.666	−0.042	−0.006	−0.007	−0.156^*^	0.030	0.091	−0.121	−0.075	0.171^*^	0.277^**^	0.392^**^	**(0.982)**	
13. EO	3.969	0.807	−0.094	0.132	−0.103	0.098	0.117	0.171^*^	−0.019	0.047	0.227^**^	0.336^**^	0.379^**^	0.474^**^	**(0.970)**

*N* = 220; ^†^*p* < 0.1, ^*^p < 0.05, ^**^p < 0.01, ^***^p < 0.001; ESE, Entrepreneurial self-efficacy; TCE, TMT collective efficacy; CTI, CEO-TMT interface; EO, Entrepreneurial orientation; Square root of AVEs are bolded and bracketed.

### Common method bias

To reduce common method bias, the methods of pre-precaution and post-check were adopted, including the respondent information hiding method and reverse item design method. In addition, different variables and dimensions of the same variable were separated to reduce the connection between the two. We used one of the most common methods to examine the issue of common method variance (CMV). Precisely, we used Harman (1967)’s single-factor test to evaluate CMV. The exploratory factor analysis was performed on all constructs items as a whole. The value of 25.433% generated was less, thus, the homology deviation problem was not significant in this study. This result indicates that no serious CMV exists between variables in this study. We also conducted the multicollinearity test. The generally accepted rule of thumb to judge the existence of multicollinearity is that the variance inflation factor (VIF) must be greater than 10 (Khan et al., 2020). Based on the results, the highest VIF value was 1.651 and the lowest tolerance value was 1.179. Therefore, multicollinearity does not seem to be a serious problem in our dataset.

### Descriptive statistics and correlation

Table 3 shows the descriptive statistics, mean, standard deviation, and correlations between entrepreneurial self-efficacy, TMT collective efficacy, CEO–TMT interface, and entrepreneurial orientation.

### Hypothesis testing

Hierarchical regression analysis was conducted following a similar method employed by prior studies, for instance, Yamini et al. (2020). This methodology allows researchers to enter variables orderly depending on the priorities or proposed relationships of variables (Osborne, 2000). Although multicollinearity issues were not a major concern in this study, we mean-centered the independent and moderator variables (Aiken et al., 1991). The results of the hierarchical linear regression analysis are shown in Table 4. In Model 1, we estimated only the effect of the control variable on the dependent variable. Among the control variables estimated, age, education, asset-liability ratio, enterprise age, and enterprise size had statistically significant effects on entrepreneurial orientation. CEO gender, past performance, tenure in post, and enterprise type were invalid. The explained variance of Model 1 was significant, and R^2^ was 0.152 (F change = 4.188, *p* < 0.01). To test the relationship between entrepreneurial self-efficacy and entrepreneurial orientation, the independent variable (entrepreneurial self-efficacy) was added to the regression Model 2. As demonstrated in Model 2, we had a significant positive relationship between entrepreneurial self-efficacy and entrepreneurial orientation (β = 0.313, *p* < 0.01). In Model 2, the explained variance was significant, and R^2^ was 0.243 (F change = 25.029, *p* < 0.01). TMT collective efficacy was included in Model 3 to estimate Hypothesis 1. In Model 3, the coefficient of β = 0.271, *p* < 0.01, and explained variance was significant with an R^2^ of 0.305 (F change = 18.583, *p* < 0.01). The CEO–TMT interface was further added in Model 4 to estimate Hypothesis 2. In Model 4, the coefficient of β = 0.364, *p* < 0.01 explained variance was significant, and R^2^ was 0.401 (F change = 33.048, *p* < 0.01).

**Table 4:** Results for hierarchical regression analysis.

Variables	Model 1	Model 2	Model 3	Model 4	Model 5	Model 6
**Control variables**						
Age	−0.165 (−2.235)^*^	−0.159 (−2.270)^*^	−0.170 (−2.538)^*^	−0.202 (−3.227)^***^	−0.168 (−2.827)^**^	−0.174 (−2.987)^**^
Education	0.141 (2.030)^*^	0.117 (1.769)^†^	0.133 (2.101)^*^	0.157 (2.651)^**^	0.156 (2.786)^**^	0.156 (2.852)^**^
CEO gender	−0.073 (−1.102)	−0.134 (−2.110)^*^	−0.131 (−2.149)^*^	−0.123 (−2.164)^*^	−0.131 (−2.378)^*^	−0.119 (−2.206)^*^
Past performance	0.101 (1.331)	0.100 (1.392)	0.085 (1.224)	0.180 (2.698) **	0.224 (3.513) ***	0.249 (3.980) ***
Tenure in post	0.142 (1.799)^†^	0.109 (1.446)	0.075 (1.033)	0.034 (0.505)	0.011 (0.164)	0.027 (0.433)
Asset-liability ratio	0.139 (2.124)^*^	0.138 (2.228)^*^	0.098 (1.630)	0.073 (1.300)	0.089 (1.663)^†^	0.134 (2.503)^*^
Enterprise age	−0.126 (−1.721)^†^	−0.086 (−1.233)	−0.058 (−0.868)	−0.004 (−0.062)	−0.022 (−0.370)	−0.040 (−0.684)
Enterprise type	0.055 (0.771)	0.041 (0.601)	0.040 (0.609)	0.064 (1.051)	0.058 (1.008)	0.063 (1.124)
Enterprise size	0.244 (3.542)^***^	0.193 (2.923)^**^	0.190 (2.990)^**^	0.105 (1.717)^†^	0.051 (0.859)	0.076 (1.316)
**Main variables**						
ESE		0.313 (5.003)^***^	0.224 (3.525)^***^	0.172 (2.865)^**^	0.135 (2.353)^*^	0.058 (0.964)
**Moderators**						
TCE			0.271 (4.311)^***^	0.150 (2.416)^*^	0.177 (2.996)^**^	0.122 (2.045)^*^
CTI				0.364 (5.749)^***^	0.394 (6.506)^***^	0.325 (5.204)^***^
**Interactions**						
ESE × TCE					0.101 (1.671)^†^	0.062 (1.041)
ESE × CTI					−0.185 (−3.177)^**^	−0.148 (−2.550)^*^
TCE × CTI					−0.209 (−3.483)^***^	−0.199 (−3.400)^***^
ESE × TCE × CTI						0.228 (3.421)^***^
**R**^**2**^	0.152	0.243	0.305	0.401	0.476	0.504
**ΔF**	4.188^***^	25.029^***^	18.583^***^	33.048^***^	9.724^***^	11.701^***^
**ΔR**^**2**^	0.152	0.091	0.062	0.096	0.075	0.029

^†^*p* < 0.1, ^*^p < 0.05, ^**^p < 0.01, ^***^p < 0.001; Standard errors are in parentheses.

In Model 5, to test the moderating effect of TMT collective efficacy on the relationship between entrepreneurial self-efficacy and entrepreneurial orientation as stated in Hypothesis 1, three interaction terms were added to the estimation in Model 5. From the regression results, the interaction term of entrepreneurial self-efficacy and TMT collective efficacy had a significant positive relationship with entrepreneurial orientation, and the standardized regression coefficient was 0.101 (*p* < 0.01). This result indicated that the positive relationship between entrepreneurial self-efficacy and entrepreneurial orientation is positively moderated by TMT collective efficacy. Therefore, Hypothesis 1 is supported.

In Model 6, to test the effect of CEO–TMT interface as stated in Hypothesis 2, the three-way interaction was added to the regression analysis in Model 6. From the regression results, the three-way interaction between entrepreneurial self-efficacy, TMT collective efficacy, and CEO–TMT interface had a significant positive relationship with entrepreneurial orientation. Moreover, the standardized regression coefficient was 0.228 (*p* < 0.01), indicating that in higher CEO–TMT interface teams, higher TMT collective efficacy can enhance the positive relationship between entrepreneurial self-efficacy and entrepreneurial orientation. Therefore, Hypothesis 2 is supported. To better understand how CEO–TMT interface affects the influence of TMT collective efficacy on the relationship between entrepreneurial self-efficacy and entrepreneurial orientation.

**Figure 2:**
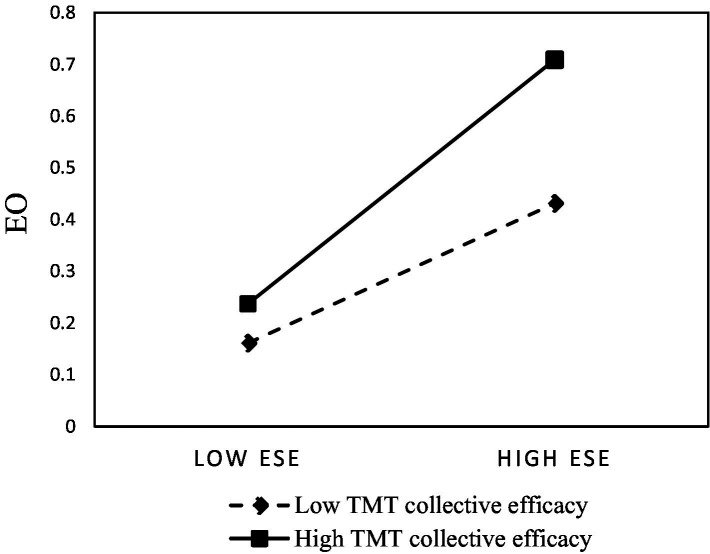
Interaction effect of entrepreneurial self-efficacy and TMT collective efficacy on entrepreneurial orientation.

**Figure 3:**
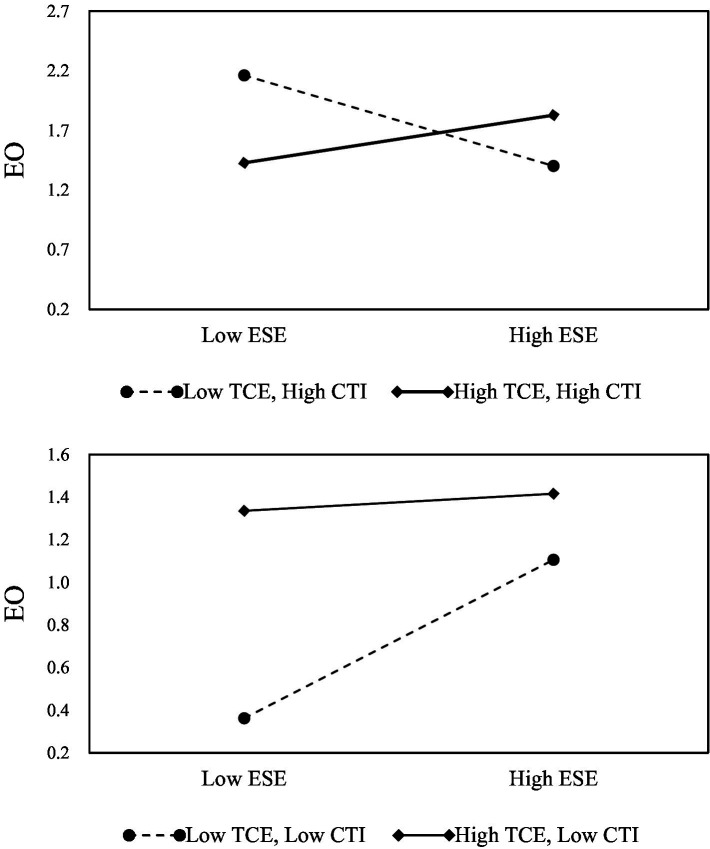
Interaction effect of CEO-TMT interface, entrepreneurial self-efficacy, and TMT collective efficacy on entrepreneurial orientation.

Finally, this research used the graphical slope plot suggested by Aiken et al. (1991). Figure 2 demonstrates the slopes for the relationship between entrepreneurial self-efficacy and TMT collective efficacy. Also, Figure 3 illustrates this interaction, reflecting, and confirming our hypothesis regarding the difference in the effect of both types of efficacy on entrepreneurial orientation depending on CEO-TMT interface.

### Robustness test

Consistent with Yamini et al. (2020), we conducted a robustness test of the model and our major results to check the validity of the data and hypotheses. Specifically, the robustness test examined the robustness of the evaluation method and index interpretation ability. To do this, we used a bootstrap test to artificially increase the data sample size to 1,000 observations. As reported in Table 5, the standard errors did not change in any meaningful way. As a result, we concluded that the results of our study are reliable.

**Table 5:** Robustness test, bootstrap test bias, and coefficients.

Variables	Model 1	Model 2	Model 3	Model 4
**Control variables**				
Age	−0.025 (0.000)^*^	−0.032 (0.000)^**^	−0.027 (−0.001)^**^	−0.027 (−0.001)^**^
Education	0.206 (0.004)^*^	0.277 (−0.002)^**^	0.276 (0.000)^**^	0.275 (−0.002)^**^
CEO gender	−0.266 (0.011)^*^	−0.244 (0.005)^*^	−0.260 (0.002)^*^	−0.236 (0.001)^*^
Past performance	0.014 (−0.001)	0.026 (0.000)^*^	0.032 (0.001)^**^	0.035 (0.001)^**^
Tenure in post	0.020 (0.002)	0.006 (0.001)	0.002 (−0.000)	0.005 (−0.000)
Asset-liability ratio	0.071 (0.004)^**^	0.038 (0.004)^†^	0.046 (0.004)^*^	0.069 (0.004)^*^
Enterprise age	−0.028 (0.003)	−0.001 (0.003)	−0.007 (0.003)	−0.013 (0.002)
Enterprise type	0.082 (0.006)	0.128 (0.013)	0.116 (−0.000)	0.126 (−0.002)
Enterprise size	0.136 (−0.000)^*^	0.074 (−0.001)	0.036 (−0.002)	0.054 (−0.001)
**Main variables**				
ESE	0.472 (−0.005)^**^	0.258 (−0.003)^*^	0.203 (0.003)^*^	0.087 (−0.001)
**Moderators**				
TCE		0.233 (0.000)^*^	0.275 (0.014)^**^	0.190 (0.015)^*^
CTI		0.441 (0.003)^**^	0.477 (−0.013)^**^	0.393 (−0.013)^**^
**Interactions**				
ESE × TCE			−0.147 (−0.009)^*^	−0.140 (−0.010)^*^
ESE × CTI			0.094 (0.002)	0.058 (0.001)
TCE × CTI			−0.152 (−0.002)^**^	−0.121 (0.001)^*^
ESE × TCE × CTI				0.175 (−0.001)^**^
Number	1,000	1,000	1,000	1,000

^†^*p* < 0.1, ^*^p < 0.05, ^**^p < 0.01, ^***^p < 0.001; Standard errors are in parentheses.

## Discussion and conclusion

The relationship between entrepreneurial self-efficacy and entrepreneurial orientation is of great interest to researchers and practitioners as it significantly dictates the course of enterprises’ decisions and entrepreneurial activities (Tasavori et al., 2018; Sellappan and Shanmugam, 2020). The literature holds a long-standing argument between different streams of studies with evidence of positive (Seet et al., 2020) and negative connections (Markman et al., 2002) between entrepreneurial self-efficacy and entrepreneurial orientation. However, prior studies lack insights into the conditions and consequences that underpin either finding. Reflecting on the essence of entrepreneurial orientation, our research joins the stream of studies on the positive relationship between entrepreneurial self-efficacy and entrepreneurial orientation with a key aim to show how the positive connection between the concepts is strengthened. Accordingly, our conceptualization argued on the moderating effects of TMT collective efficacy and CEO-TMT interface on prior assertion of their connection power and beneficial outcomes (Chen et al., 2019). In this light, our proposal sought to address the research question: How does entrepreneurial self-efficacy affect entrepreneurial orientation?

We estimated the proposed positive moderating effect of TMT collective efficacy (Luo and Lin, 2022) and CEO-TMT interface (Georgakakis et al., 2022) as discussed in Hypotheses 1 and 2, respectively. Contrary to our expectations, our findings showed positive and negative effects. First, we found that entrepreneurial self-efficacy positively affects entrepreneurial orientation, thereby enriching prior assertions of their positive linkage (Hwan, 2020). Second, our results showed that TMT collective efficacy positively strengthens the positive relationship between entrepreneurial self-efficacy and entrepreneurial orientation. The finding validates our claim on the need to reinforce the entrepreneurial self-efficacy and entrepreneurial orientation positive nexus. By this means, we point out to enterprises the alleyway or conditions under which the reinforcement occurs. Specifically, we underscore TMT collective efficacy as an efficient team-level component that helps an enterprise to take pioneering actions to enhance their enterprise’s active competition, innovation, and risk-taking entrepreneurial-oriented practices. Thus, we shed additional light on prior findings regarding the critical role of TMTs in enterprise goal attainment (Luo and Lin, 2022). On this account, we can say TMT collective efficacy expedites an enterprise’s entrepreneurial self-efficacy and consequently contributes to the enterprise’s entrepreneurial orientation. Prior studies, for example, Zhong et al. (2022) highlighted that the CEO plays a crucial role in determining strategic direction and setting policies. In this regard, our findings put forward TMT collective efficacy as an indispensable human capital that possesses the collaborative capability to complement a CEO’s entrepreneurial self-efficacy and that of his or her enterprise.

Finally, our study further assessed the effect of CEO-TMT interface (Georgakakis et al., 2016) on the moderating effect of TMT collective efficacy on the relationship between entrepreneurial self-efficacy and entrepreneurial orientation. We found a negative but significant moderating effect when CEO–TMT interface interacts with TMT collective efficacy (two-way interaction). However, the moderating effect becomes positive when CEO-TMT interface interacts with both entrepreneurial self-efficacy and TMT collective efficacy (three-way interaction). The negative finding infers that a stronger negative moderating effect exists between CEO-TMT interface and TMT collective efficacy, as a result, dampens or decreases the indirect positive effect of TMT collective efficacy on entrepreneurial orientation. In other words, in the absence of CEO entrepreneurial efficacy, greater levels of CEO-TMT interface weakness TMT collective efficacy’s beneficial impact on entrepreneurial orientation. On the other hand, CEO-TMT interface positively promotes entrepreneurial orientation when entrepreneurial self-efficacy and TMT collective efficacy co-exist between an enterprise’s CEO and TMT. Although the positive finding obtained supports this study’s Hypothesis 2, the discovery of the negative interaction effect adds fresh insight (caution signals) regarding the factors whose interplay counter-attacks enterprises’ efforts to reinforce their entrepreneurial self-efficacy and entrepreneurial orientation nexus. In a nutshell, our findings enrich prior studies on CEO-TMT interface (Georgakakis et al., 2016) and emphasize how effective collaboration or otherwise between CEO and TMT members develop or thwart their individual and group level competencies and how noticeable barriers to shared ability, decision, and task executive for the benefit of their entrepreneurial orientation can be addressed. By far, no study has unveiled this essential knowledge and the differential ways (positive and negative) in which it unfolds.

This study offers two novel theoretical implications. First, our study provides the possibility to build and test how individual-level entrepreneurial self-efficacy and group-level TMT collective efficacy interact (exchanges), which is an understudied issue in social cognitive theory. In addition, scholars grounded the link between entrepreneurial self-efficacy and entrepreneurial orientation using different theoretical lenses, resulting in several conclusions with no insights into their advancement *via* social cognitive theory (SCT). By employing SCT (Seet et al., 2020), we show that individual exchanges depend on collaborative capabilities that advance individual and group-level cognitive features. By exploring the moderating effect of TMT collective efficacy and CEO-TMT interface, we add to SCT the specific factors that underlie advancing early discoveries of the positive connection between entrepreneurial self-efficacy and entrepreneurial orientation and how these moderating factors cause negative occurrences between them. In this vein, we validate SCT as a solid basis for understanding CEO and TMT exchanges that expedite individual and group-level capabilities such as efficacy (Alavi and McCormick, 2018; Donohoo et al., 2018).

Second, existing research based on SCT found both positive significant (Hwan, 2020) and no significant effects (Chandler and Jansen, 1997) between entrepreneurial self-efficacy and entrepreneurial-oriented behaviors. As such, conclusions were based on its variation across contexts. This study’s examination represents two conditional effects of CEO-TMT interface. First, we enrich prior findings based on SCT (Seet et al., 2020), by demonstrating that there is a positive significant effect on entrepreneurial orientation when a team-level factor (CEO-TMT interface) interacts with other team and individual-level variables (TMT collective efficacy and entrepreneurial efficacy). However, in the absence of these exchanges between work incumbents/members, a negative exchange is initiated which hurt the enterprise’s entrepreneurial orientation. This finding does not only contribute to addressing the lack of theoretical evidence on the conditions that account for the varied or inconsistent relationship between entrepreneurial self-efficacy and entrepreneurial orientation but can also aid scholars and practitioners to understand why such occurrences exist and how they can be controlled.

### Practical implications

This study also provides important practical implications for enterprises and their top management teams. First, our findings on the positive relationships between entrepreneurial self-efficacy, TMT collective efficacy, and entrepreneurial orientation serve as a guide for CEOs to exercise control while providing support for their TMTs to accomplish enterprise entrepreneurial orientation objectives. In this light, for example, the CEO can provide more intrinsic motivation such as inspirational incentives and personalized care to improve TMT collective efficacy. In addition, CEOs need to acknowledge or view TMT members as important strategic resources with task execution propensities that complement a CEO’s attributes for enterprise entrepreneurial orientation development rather than a sole focus on a CEO’s value over that of TMTs. Prior studies’ practical implications shed light on the duty of CEOs in establishing a conducive work environment. Precisely, in the field of entrepreneurship, the CEO’s confidence or conviction affects his/her environment, as a result, aids work incumbents to achieve goals such as the documented outcomes of entrepreneurial behavior (Chen et al., 1998; Hand et al., 2020). Consequently, there is a need for unrelenting cooperation, effective communication, and periodic intra-enterprise training on task execution to enhance individual-to-individual level exchanges and augment team-level efficacies for the cultivation and performance of enterprise entrepreneurial orientation.

Second, reflecting on the positive and negative influence of CEO–TMT interface, this study provides the following standards for enterprises to build and optimize the benefits of CEO-TMTs as well as mitigate the negative consequence of retrogression in collective efficacy and entrepreneurial orientation. Thus, CEOs should not compromise on the resource pool of TMT candidates in their recruitment and selection. For instance, TMT candidates’ past working experiences, skills, and abilities should be the topmost criteria for external and internal recruitment and selection. This implies that CEOs should partake in recruiting TMTs and ensure there is a cognitive similarity between incoming TMT member (s) and the incumbent CEO to reduce possible conflicts that arise in collaboration (TMTs and CEOs). Driven along with the emphasis, the CEO could foster a highly ethical and inclusive work environment for the sustainable development of the TMT members’ efficacy. Cumulatively, these practical implications would serve as a guiding mechanism for effective job crafting for the efficient operation of the CEO and TMT to attain a long-term impact on their entrepreneurial orientation.

### Limitations and suggestions for future research

This study also has limitations that need to be further addressed in subsequent studies. Firstly, this study takes entrepreneurial enterprises in the context of China as its research object to test its hypothesis. As our subject, China meets all the requirements of our study, and the results strongly support our hypothesis. However, future research can consider the difference in the impact of entrepreneurial self-efficacy on entrepreneurial orientation in trans-national and trans-cultural contexts. Also, researchers can further carry out cross-cultural comparisons in multiple countries to clarify the difference in entrepreneurial self-efficacy in different cultural contexts. Doing so could provide results that could be more generalized.

Secondly, although the empirical study uses cross-sectional data and verifies the validity and reliability of its method, researchers could use other data types to extend the empirical contribution of this study. Nevertheless, the cross-sectional data are suitable and also consistent with similar investigations in the literature (Ma et al., 2022). Similarly, considering the different stages of an enterprise’s life cycle, the management decisions have different emphases which influence the entrepreneurial orientation level of alienation (Kesidou and Carter, 2018). As a result, future studies can explore the impact of entrepreneurial self-efficacy on entrepreneurial orientation through longitudinal sequence data or panel data.

Lastly, further exploration can be made based on our conceptual model to provide new perspectives on other crucial external mechanisms for the development of entrepreneurial self-efficacy and entrepreneurial orientation.

## Data availability statement

The raw data supporting the conclusions of this article will be made available by the authors, without undue reservation.

## Ethics statement

Ethical review and approval was not required for the study on human participants in accordance with the local legislation and institutional requirements. The patients/participants provided their written informed consent to participate in this study. Written informed consent was obtained from the individual (s) for the publication of any potentially identifiable images or data included in this article.

## Author contributions

XP and XS contributed to the conception and design of the study. XS and EH organized the database, data collection, and conducted the statistical analysis. XS wrote the first draft and handled the final corrections after proofreading by XP and EH. XS and EH wrote the methodology section under the guidance of XP. All authors contributed to the article and approved the submitted version.

## Funding

This work was supported by the University of Science and Technology of China (USTC) Funding for Featured Liberal Arts under grant no. YD2160002003, University of Science and Technology of China (USTC) introduction of talents for scientific research and startup special fund project under grant no. KY2160000003, and Anhui Province Philosophy and Social Science Planning Project under grant no. AHSKY2019D025.

## Conflict of interest

The authors declare that the research was conducted in the absence of any commercial or financial relationships that could be construed as a potential conflict of interest.

## Publisher’s note

All claims expressed in this article are solely those of the authors and do not necessarily represent those of their affiliated organizations, or those of the publisher, the editors and the reviewers. Any product that may be evaluated in this article, or claim that may be made by its manufacturer, is not guaranteed or endorsed by the publisher.
